# The gene signature linked to lactate metabolism predicts the prognosis and correlates with the immune status of head and neck squamous cell carcinoma

**DOI:** 10.3389/fgene.2025.1540841

**Published:** 2025-04-04

**Authors:** Jian Xiao, Wei Li, Guolin Tan, Ru Gao

**Affiliations:** Department of Otolaryngology-Head and Neck Surgery, The Third Xiangya Hospital of Central South University, Changsha, Hunan, China

**Keywords:** head and neck squamous cell carcinoma, lactic acid metabolism score, prognostic risk model, risk score, immune

## Abstract

Lactate, traditionally viewed as a byproduct of glycolysis, is increasingly recognized as a pivotal regulatory factor in cancer biology. This study addresses the limited understanding of lactate metabolism-related genes in head and neck squamous cell carcinoma (HNSC) by constructing a prognostic risk model centered on these genes to enhance prediction and treatment strategies for HNSC. Utilizing the Lactate Metabolism score (LMs) derived from The Cancer Genome Atlas (TCGA), we identified five key genes significantly associated with prognosis in HNSC patients. These genes were integrated into a prognostic risk model developed through Cox regression analysis, which demonstrated superior predictive performance, achieving area under the curve (AUC) values greater than 0.8 for five-year survival. The risk scores generated by our model were significantly correlated with critical features of the tumor microenvironment, including immune characteristics and markers of immune evasion. Higher risk scores correlated with a more tumor-promoting microenvironment and increased immune suppression, underscoring the model’s relevance in understanding HNSC progression. Additionally, eight critical hub genes were identified, revealing significant differences in gene expression between risk score groups. Functional analyses demonstrated that the low-risk group exhibited a more favorable prognosis and enhanced immune characteristics. Our findings suggest that the lactate metabolism-based prognostic model may have implications for guiding the development of personalized treatment approaches, as it highlights the potential for targeted interventions that could modulate the tumor microenvironment and immune response.

## Introduction

Head and neck squamous cell carcinoma (HNSC) ranks as the sixth most prevalent cancer globally with 890,000 new cases and 450,000 deaths reported in 2018 (Ferlay et al., 2019; Bray et al., 2020). The incidence of HNSC is projected to escalate by 30%, translating to approximately 1.08 million new cases annually, by 2030, as forecasted by the Global Cancer Observatory (Ferlay et al., 2019; Bray et al., 2020). Despite significant advancements in diagnosis, prognosis, and treatment of HNSC, the clinical outcomes have shown little improvement over the past few decades (Pulte and Brenner, 2010). In addition, the suicide rate among HNSC survivors is the second highest among all cancer survivors, potentially linked to diminished quality of life and heightened psychological distress (Osazuwa-Peters et al., 2018). Hence, it is necessary to investigate further the molecular mechanisms underlying the progression of HNSC to improve patient treatment and prognosis.

Lactic acid, an abundant metabolite in the human circulatory system, was previously regarded as a byproduct of glycolysis (Psychogios et al., 2011). However, it has emerged as a pivotal regulator in the progression, maintenance, and metastasis of cancer (Doherty and Cleveland, 2013). Elevated levels of lactate in tumors have been associated with increased metastatic potential, tumor recurrence, and poor prognosis (Apostolova and Pearce, 2022; Feng et al., 2020). Despite these insights, there remains a paucity of research dedicated to elucidating the role of lactate metabolism in HNSC.

Traditional treatment modalities for head and neck squamous cell carcinoma (HNSC), including surgery, radiation therapy, and chemotherapy, have faced persistent challenges in improving the prognosis for patients with advanced or recurrent disease (Pulte and Brenner, 2010). In recent years, the therapeutic landscape for HNSC has been revolutionized by the introduction of targeted therapies and immunotherapies (Bhatia and Burtness, 2023). Targeted therapies, such as inhibitors of the epidermal growth factor receptor (EGFR), have shown promise in patients with EGFR-overexpressing tumors. Immunotherapeutic strategies, including the use of immune checkpoint inhibitors like pembrolizumab and nivolumab, have demonstrated efficacy in specific subsets of HNSC patients, particularly those with high tumor mutational burden or microsatellite instability (Vos et al., 2021; Liu et al., 2023). The elucidation of lactate metabolism-related genes in HNSC provides valuable insights into the tumor microenvironment and its interaction with therapeutic agents. By integrating these innovative treatment approaches into our research on lactate metabolism-related genes, we aim to contribute to the development of more effective and personalized treatment strategies for HNSC patients.

In this study, we conducted a comprehensive evaluation of the expression profile of Lactate Metabolism-Related Genes (LMRGs) in HNSC. Initially, we identified key genes that hold prognostic significance from a cohort of 546 HNSC patients, based on their expression levels of LMRGs. These patients were subsequently categorized into two distinct subtypes. Next, we constructed a prognostic model utilizing the Lactate Metabolism score (LMs), which facilitated the identification of pivotal genes. Moreover, we investigated the correlation between LMs and various factors such as immune infiltration, mutation, copy number variation (CNV), and responsiveness to immunotherapy.

## Results

### Expression and Prognosis of LMRGs in HNSC

**FIGURE 1:**
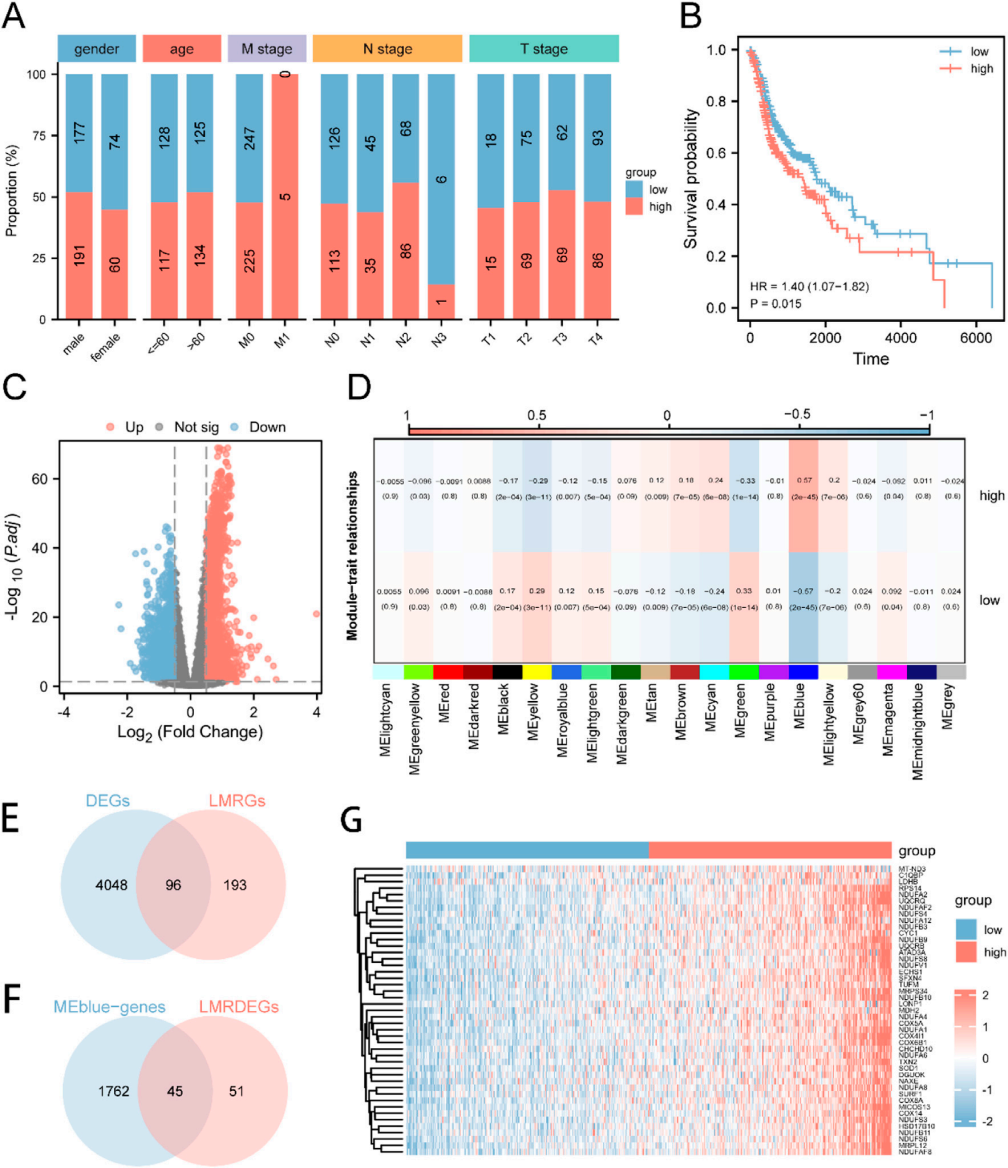
Expression and Prognosis of LMRGs in HNSC **(A)** The patient population was divided into two groups, namely, LMs-low and LMs-high, based on gender, age, M stage, N stage, and T stage proportions. **(B)** A Kaplan-Meier survival curve was generated to compare the survival rates of the LMs-low and LMs-high groups. **(C)** A volcano plot was utilized to highlight the variance in gene expression between the LMs-low and LMs-high groups. **(D)** Heatmap illustrating the correlation between gene modules identified by WGCNA and the LMs-low and LMs-high groups. **(E)** Venn diagram demonstrating the intersection between DEGs and LMRGs. **(F)** Venn diagram displaying the overlap between LMRDEGs and genes in the MEblue module. **(G)** A heatmap was generated to present the differential expression patterns of key genes observed between the LMs-low and LMs-high groups.

The workflow and standardization of the dataset in our study are depicted in Supplementary Figure S1. We compared the expression levels of 289 LMRGs across the TCGA-HNSC cohort, with detailed results summarized in Supplementary Table S1. Employing a correlation algorithm, we analyzed the LMs for each HNSC patient. Based on the median LMs value, patients were stratified into two distinct groups: LMs-high and LMs-low. We examined the correlation between the LMs status and clinical variables (such as gender, age, M stage, N stage, and T stage). Our analysis revealed no significant associations between LMs grouping and gender, age, or T stage. However, intriguing correlations were observed with the M stage and N stage, as depicted in Figure 1A. Notably, LMs-high patients were predominantly observed in the M1 stage and exhibited a higher proportion in the N2 stage compared to LMs-low patients. Furthermore, we generated Kaplan-Meier curves for the LMs-high and LMs-low groups (Figure 1B). These curves revealed a significant difference in overall survival, with patients in the LMs-low group exhibiting a more favorable prognosis compared to those in the LMs-high group (p = 0.015).

Moreover, we conducted an investigation to identify genes that were differentially expressed (DEGs) between the LMs-low and LMs-high groups. The results of this analysis are vividly depicted in the volcano plot (Figure 1C), which highlights 2334 genes that were significantly upregulated and 1810 genes that were downregulated in the LMs-high group compared to the LMs-low group. To perform Weighted Gene Co-expression Network Analysis (WGCNA), we identified 20 gene modules, among which the MEblue module exhibited the highest correlation with LMs (Figure 1D). We then carried out an intersection analysis between DEGs from the TCGA-HNSC dataset and LMRGs, resulting in 96 LMRDEGs (refer to Figure 1E). Subsequently, we performed another intersection analysis between LMRDEGs and MEblue genes, leading to the identification of 45 key genes (Figure 1F; Supplementary Table S2). To visually represent the differential expression of these key genes between the LMs-low and LMs-high groups, we generated a heatmap (Figure 1G).

Furthermore, we conducted a comparative analysis of the expression levels of critical genes across three distinct datasets: TCGA-HNSC, GSE6631, and GSE107591. In the TCGA-HNSC dataset, we observed a substantial increase in the expression of 45 key genes in the LMs-high group compared to the LMs-low group. Similarly, within the GSE6631 dataset, the LMs-high group exhibited significantly higher expression of 11 genes (C1QBP, COX4I1, COX5A, COX6B1, LDHB, NDUFA2, NDUFB3, NDUFS3, NDUFS4, SOD1, UQCRB) when compared to the LMs-low group. Likewise, in the GSE107591 dataset, 12 genes (C1QBP, COX4AI1, CYC1, MRPS34, NDUFA12, NDUFA6, NDUFA8, NDUF4F2, NDUFB9, NDUFV1, SOD1, TXN2) displayed significantly increased expression in the LMs-high group relative to the LMs-low group (Supplementary Figure S2).

### Selection and molecular subtyping of prognostic-related key genes

**FIGURE 2:**
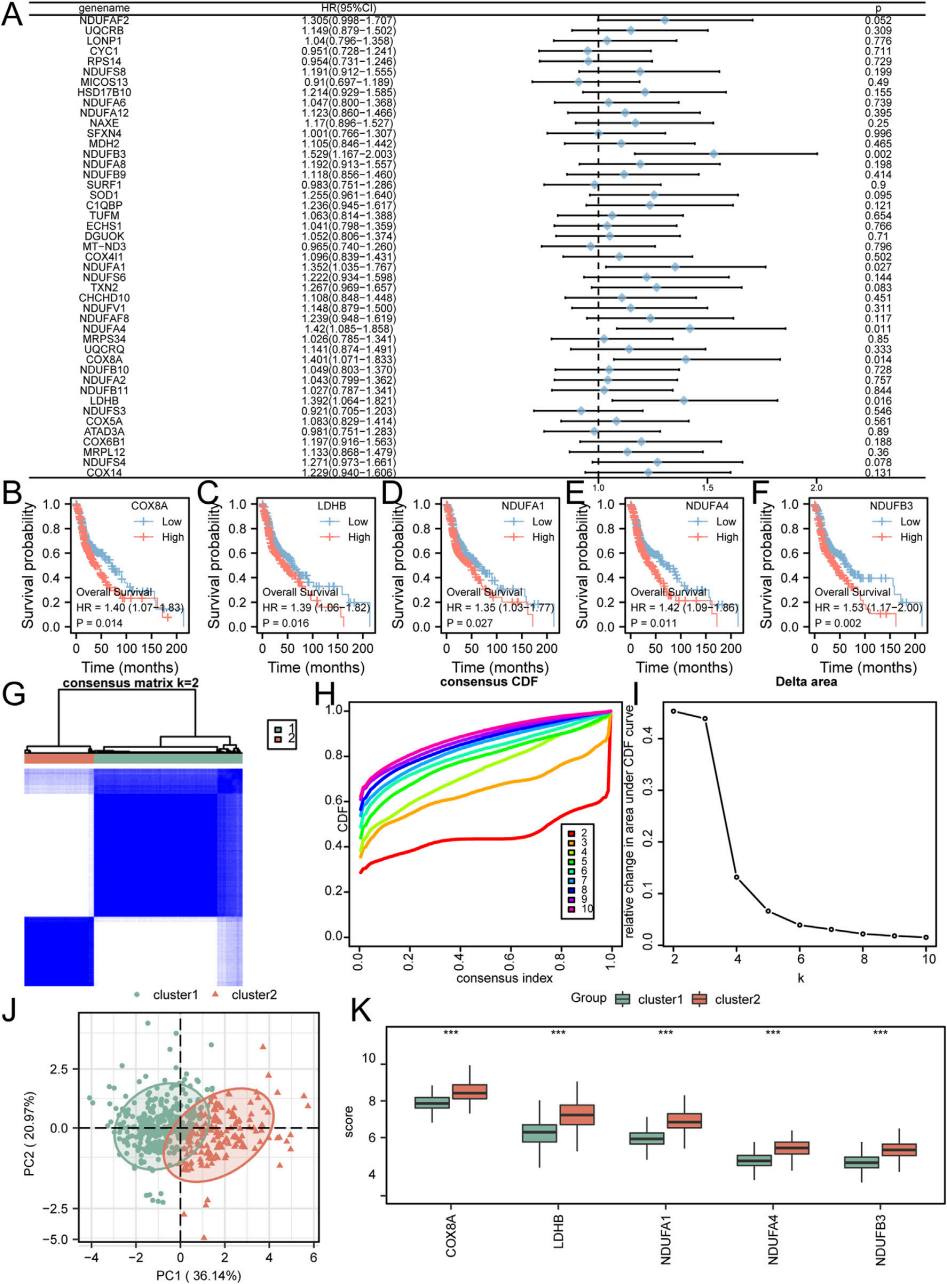
Selection of Prognostic-Related Key Genes and Molecular Subtyping **(A)** Key genes were analyzed using univariate Cox regression, as shown in the forest plot. B-F. Kaplan-Meier curves were plotted for COX8A **(B)**, LDHB **(C)**, NDUFA1 **(D)**, NDUFA4 **(E)**, and NDUFB3 **(F)**. **(G–I)** HNSC samples were clustered into two categories using unsupervised clustering **(G)**; Corresponding CDF plot **(H)** and Delta Area plot **(I)** were generated. **(J)**. PCA plot was constructed to visualize the differentiation between cluster 1 and cluster 2. **(K)** The comparative grouping plot illustrated the distinct grouping patterns of key genes within Cluster 1 and Cluster 2. *P < 0.05; **P < 0.01; ***P < 0.001.

The initial investigation focused on evaluating the prognostic significance of 45 crucial genes in HNSC patients. This assessment was achieved through the utilization of univariate Cox regression (Figure 2A) and Kaplan-Meier analysis to establish the association between gene expression and molecular subtypes. Employing a screening threshold of P < 0.05, we identified COX8A (Figure 2B), LDHB (Figure 2C), NDUFA1 (Figure 2D), NDUFA4 (Figure 2E), and NDUFB3 (Figure 2F) as key genes associated with prognosis. Subsequently, we employed unsupervised consensus clustering to explore the underlying structure of the HNSC patient cohort. This analysis revealed strong intragroup correlations and low intergroup correlations when k = 2. This finding suggests that HNSC patients can be categorized into two distinct subtypes: cluster 1 (n = 342) and cluster 2 (n = 160) (Figures 2G–I). Notably, principal component analysis (PCA) exhibited significant disparities in lactic acid metabolism-related transcriptome profiles between cluster 1 and cluster 2 (Figure 2J). Furthermore, we examined the distribution of the five key genes (COX8A, LDHB, NDUFA1, NDUFA4, NDUFB3) across the different immune feature subtypes identified by consensus clustering. Significant variations in the expression patterns of these genes were observed between cluster 1 and cluster 2 (Figure 2K).

### Analysis of gene mutations in key genes and construction of a risk model related to lactate metabolism

**FIGURE 3:**
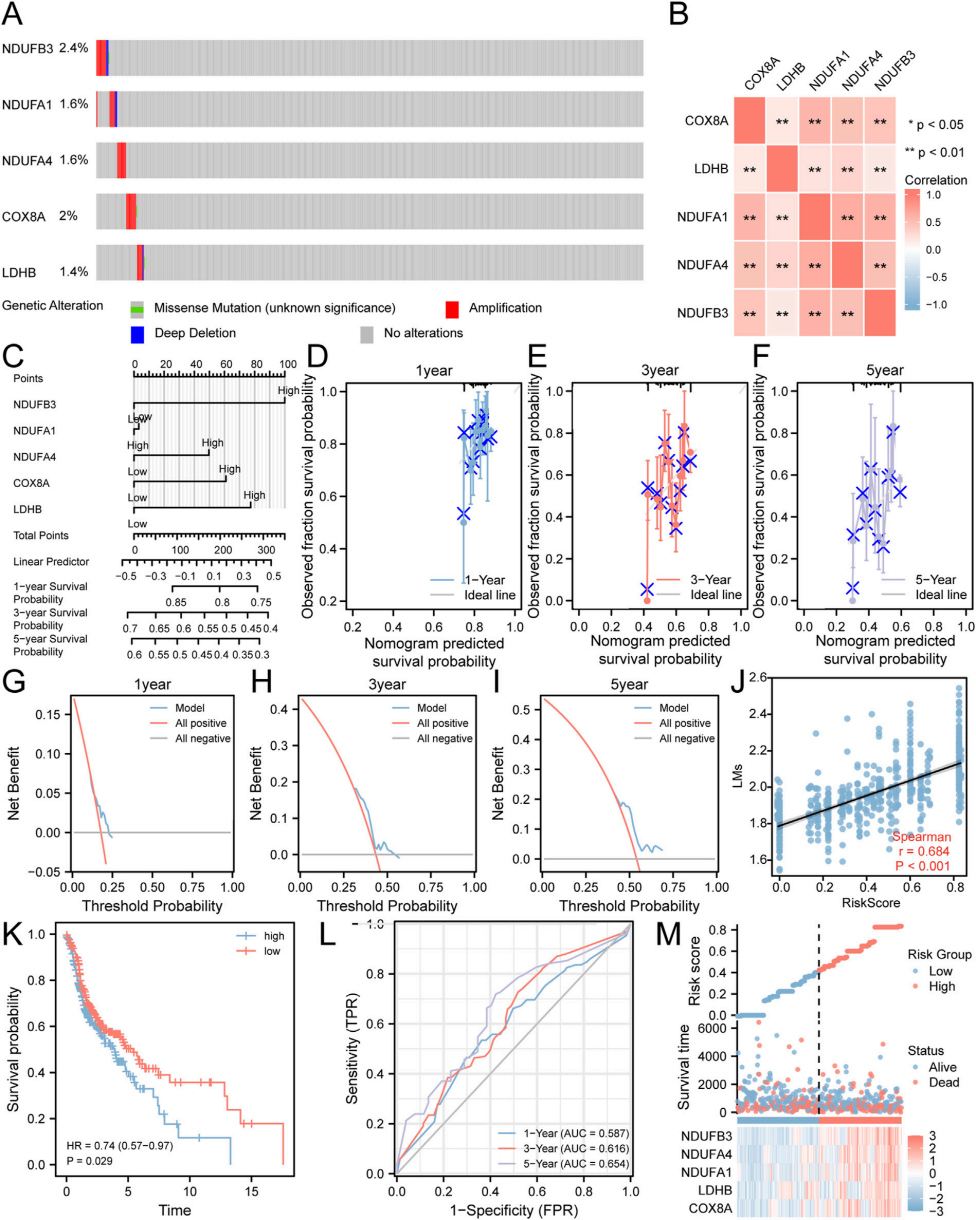
Risk Model Construction Related to Lactate Metabolism. **(A)** Analysis of gene mutations in the TCGA-HNSC dataset for the five key genes. **(B)** Heatmap illustrating the correlation among the five key genes. **(C)** A nomogram representing the Cox regression model. **(D–F)** Calibration curves plotting the results from prognostic nomogram analyses of the Cox regression model at one-year **(D)**, three-year **(E)**, and five-year **(F)** intervals. **(G–I)** Decision curve analysis plots for the prognostic Cox regression models at one-year **(G)**, three-year **(H)**, and five-year **(I)** time points. **(J)** Scatter plot demonstrating the correlation between the risk score derived from the Cox regression model and LMs. **(K)** Kaplan-Meier curve displaying the impact of the risk score derived from the Cox regression model on patient prognosis. **(L)** Time-dependent ROC curve illustrating the predictive ability of the risk score derived from the Cox regression model for the TCGA-HNSC dataset. **(M)** Plot depicting the risk factors based on the Cox regression model. **P < 0.01.

We analyzed gene mutations in five key genes (COX8A, LDHB, NDUFA1, NDUFA4, NDUFB3) using samples from the TCGA-HNSC dataset and the cBioPortal database. The findings (Figure 3A) showed that NDUFB3 had the highest total mutation count, accounting for 2.4% of the total samples in the TCGA-HNSC dataset, mainly presenting as significant amplification. We also observed a significantly high correlation (p < 0.01) for all pairwise combinations of the 5 key genes (Figure 3B).

To further investigate their prognostic significance in HNSC, these five key genes were subjected to multivariate Cox regression analysis (Figure 3C; Supplementary Table S3). The results demonstrated that patients with elevated expression levels of COX8A, LDHB, NDUFA4, and NDUFB3 had a poorer prognosis. Furthermore, we conducted prognostic calibration analysis at discrete time points: one-year (Figure 3D), three-year (Figure 3E), and five-year (Figure 3F). These analyses utilized the nomogram derived from the multivariate Cox regression model to generate corresponding calibration curves (Figures 3D–F). Notably, our DCA results indicated that the clinical predictive effect of the constructed Cox regression prognostic model was most pronounced at the five-year mark, followed by the three-year and one-year intervals (Figures 3G–I).

In order to assess the correlation between the risk score of the Cox model and the LMs, scatter plots were generated, revealing a moderate correlation (r = 0.684) (Figure 3J). Moreover, the KM curve (Figure 3K) demonstrated a worse prognosis for HNSC patients with high-risk scores compared to those with low-risk scores. Additionally, the ROC curve (Figure 3L) showed that the Cox risk score had a certain predictive ability for the overall survival of HNSC patients, with improved prediction as time increased. Finally, we visualized the distribution of lactate metabolism-related risk scores among HNSC patients by generating a heatmap based on the Cox risk scores (Figure 3M).

### Differential expression analysis and GO and KEGG analyses based on Cox risk score groups

**FIGURE 4:**
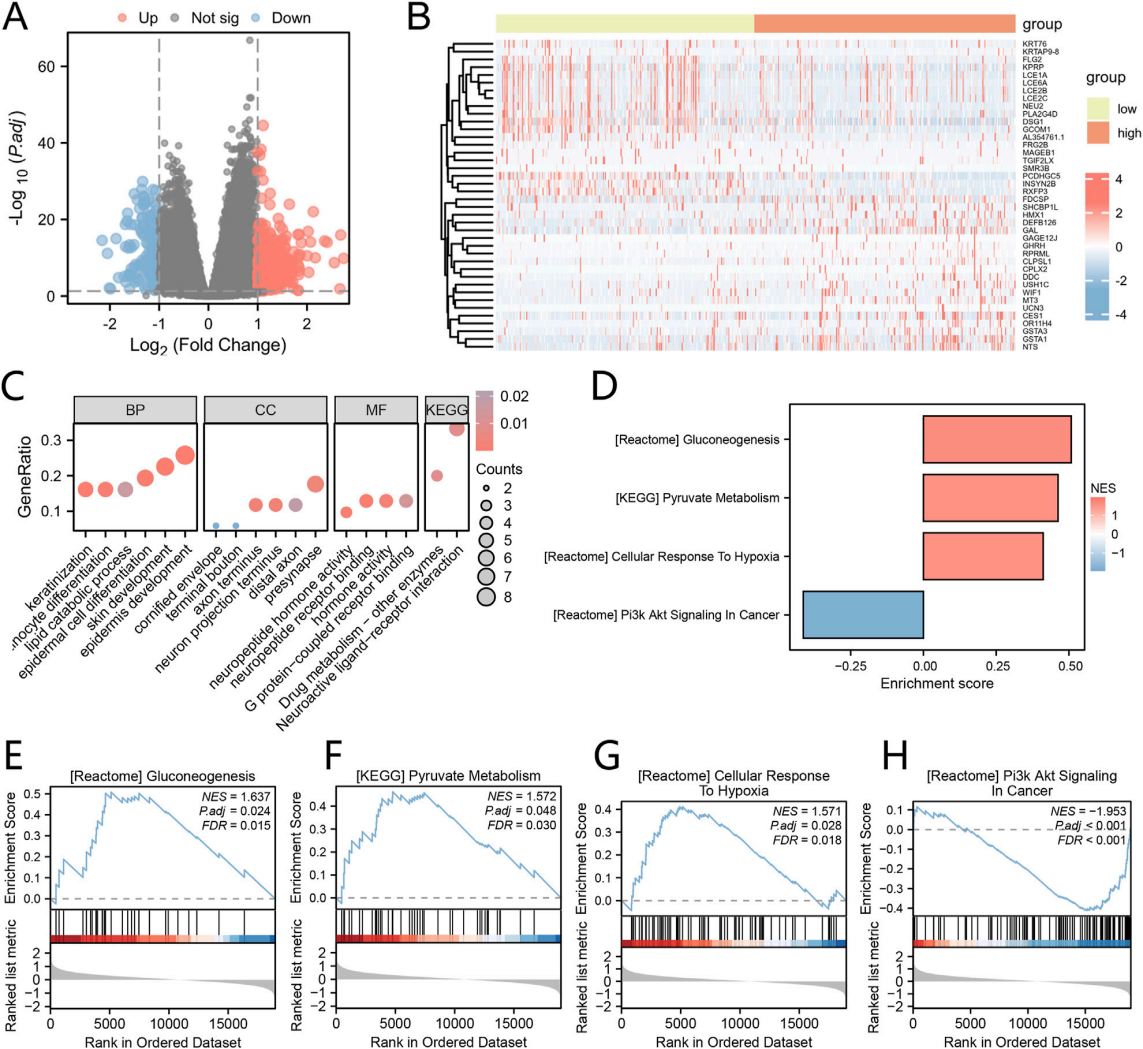
Analysis of Differential Expression and GO/KEGG Based on Cox Risk Score Groups **(A)** The Volcano plot displays the DEGs between groups with high and low riskscores, as determined by the Cox regression model. **(B)** The Heat map illustrates the DEGs between groups with high and low riskscores, as determined by the Cox regression model. **(C)** The bubble plots present the results of the enrichment analyses for GO and KEGG pathways in the DEGs. **(D)** The Barplot showcases the GSEA. E-H. Significant enrichment of DEGs in the Gluconeogenesis **(E)**, Pyruvate metabolism **(F)**, Cellular Response to Hypoxia **(G)**, and PI3K/AKT Signaling in Cancer **(H)** pathways. *P < 0.05; **P < 0.01; ***P < 0.001.

The TCGA-HNSC dataset samples were categorized into high-risk and low-risk groups based on the median Cox risk score. To identify DEGs associated with the Cox risk score, we conducted a differential expression analysis on the expression profile data of the TCGA-HNSC dataset. The DEGs for our study were selected based on the criteria of p.adjust <0.05 and the top 20 genes with positive and negative log fold change (logFC). These genes included GHRH, GSTA1, GAGE12J, UCN3, WIF1, CPLX2, NTS, DDC, CES1, SHCBP1L, MT3, FDCSP, HMX1, USH1C, RPRML, GAL, CLPSL1, OR11H4, DEFB126, GSTA3, NEU2, FLG2, TGIF2LX, MAGEB1, KPRP, AL354761.1, LCE2B, PCDHGC5, KRT76, KRTAP9-8, RXFP3, GCOM1, DSG1, LCE6A, LCE1A, SMR3B, PLA2G4D, LCE2C, INSYN2B, and FRG2B. In order to visualize the results of the differential analysis, we generated a volcano plot (Figure 4A) and a heat map (Figure 4B).

To gain insights into the biological processes, molecular functions, cellular components, and biological pathways associated with these 40 DEGs, we performed GO and KEGG enrichment analyses. The GO functional enrichment analysis (Figure 4C) and KEGG enrichment analysis revealed that the 40 DEGs were significantly enriched in various biological processes, including epidermis development, skin development, epidermal cell differentiation, keratinization, keratinocyte differentiation, lipid catabolic process. In terms of cellular components, they were associated with presynapse, axon terminus, neuron projection terminus, distal axon, cornified envelope, and terminal bouton. The molecular functions identified neuropeptide receptor binding, hormone activity, G protein-coupled receptor binding, and neuropeptide hormone activity. Additionally, the pathways identified were Neuroactive ligand-receptor interaction and Drug metabolism - other enzymes. (Supplementary Table S4).

To evaluate the impact of gene expression levels on the categorization of high and low risk score groups as determined by the Cox regression model, we conducted Gene Set Enrichment Analysis (GSEA). The GSEA results revealed significant enrichment of DEGs in various pathways (Figure 4D). Notably, these pathways included Gluconeogenesis (Figure 4E), Pyruvate metabolism (Figure 4F), Cellular Response to Hypoxia (Figure 4G), and PI3K/AKT Signaling in Cancer (Figure 4H), among others, within the TCGA-HNSC dataset (Supplementary Table S5).

Utilizing the STRING database, we elucidated the interactions among differentially expressed genes (Supplementary Figure S3A). Subsequently, we identified central genes by intersecting the highest-ranked genes obtained from five disparate algorithms (Degree, MNC, MCC, EPC, and Closeness), as shown in Supplementary Figure S3B. Moreover, the GeneMANIA algorithm was employed to visualize the interaction network of genes functionally associated (Supplementary Figure S3D). In addition, we predicted the miRNA interactions for the eight central genes, as depicted in Supplementary Figure S3C, which led to the construction of an mRNA-miRNA interaction network. This network consists of six central genes (NTS, GHRH, LCE2B, DDC, PCDHGC5, and MT3) and 53 miRNA molecules, encompassing a total of 54 mRNA-miRNA interaction pairs. (Supplementary Figure S3C; Supplementary Table S6).

### Analysis of immune infiltration and scoring in the TCGA-HNSC dataset

**FIGURE 5:**
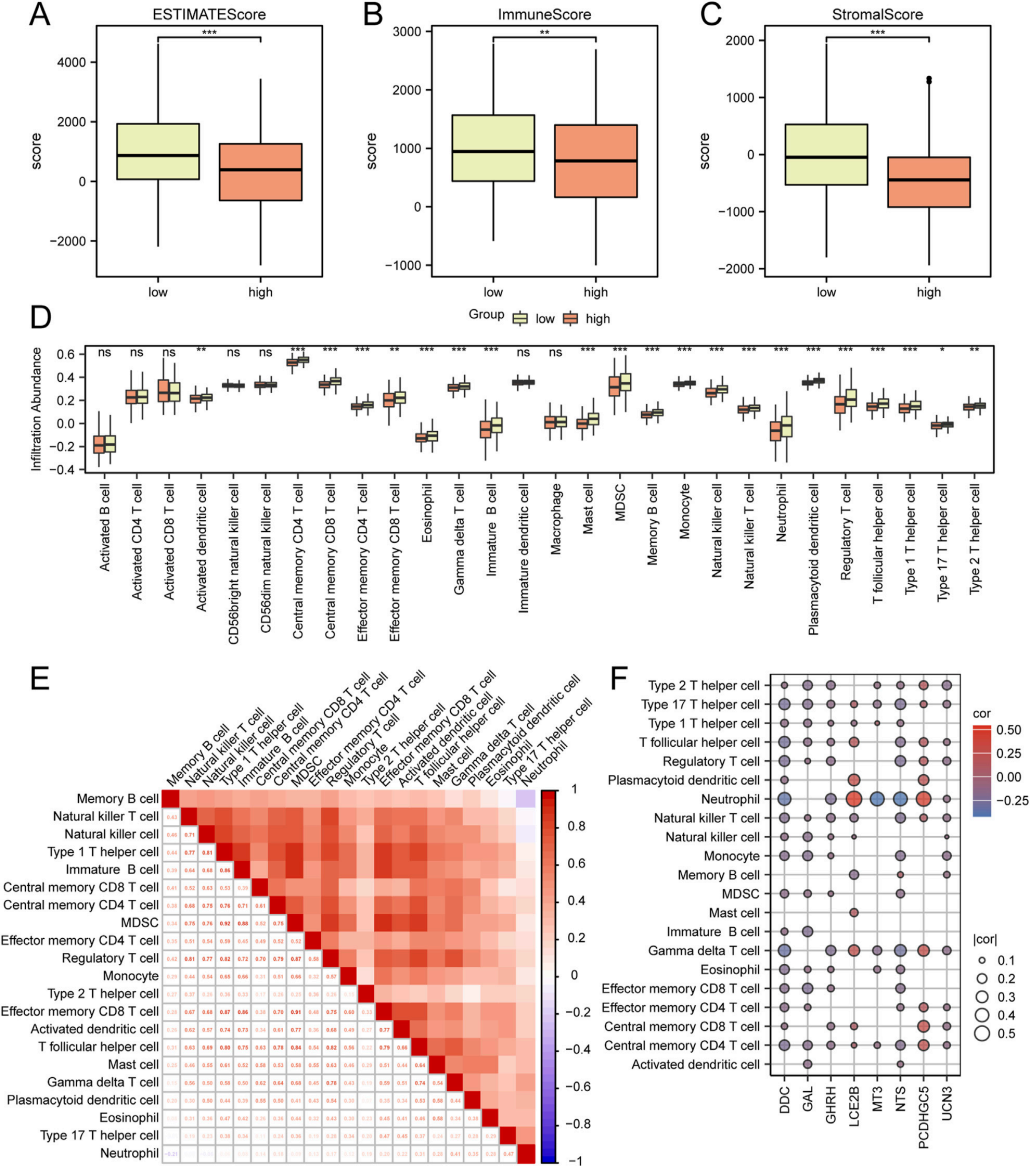
Analysis of Immune Scoring and Immune Infiltration in the TCGA-HNSC Dataset **(A–C)** Comparative analysis of ESTIMATE scores, Immune scores, and Stromal scores among the low and high riskscore groups in the TCGA-HNSC dataset. **(D)** Comparative assessment of immune cell infiltration between the low and high riskscore groups in the TCGA-HNSC dataset. **(E)** Heat map depicting the correlation among different immune cell populations. **(F)** Heat map illustrating the correlation between immune cells and hub genes. *P < 0.05; **P < 0.01; ***P < 0.001.

In order to evaluate immune infiltration and scoring in the TCGA-HNSC dataset, a range of immune and stromal scores were examined based on the Cox risk score. The findings indicated significant variations (P < 0.05) among different groups within this dataset for the ESTIMATE score (Figure 5A), Immune score (Figure 5B), and Stromal score (Figure 5C).

Subsequently, an analysis was conducted to compare immune cell infiltration between the low risk and high risk score groups as identified by Cox regression analysis (Figure 5D). Additionally, a correlation heat map was generated to assess the relationship between immune cell populations (Figure 5E). Furthermore, an evaluation of the correlation between immune cells and hub genes (GAL, NTS, GHRH, UCN3, LCE2B, DDC, PCDHGC5, MT3) was performed (Figure 5F). The results demonstrated significant differences (p < 0.05) in infiltration for 21 immune cell types (Central memory CD4 T cell, Central memory CD8 T cell, Effector memory CD4 T cell, Effector memory CD8 T cell, Gamma delta T cell, Immature B cell, Memory B cell, Regulatory T cell, T follicular helper cell, Type 1 T helper cell, Type 17 T helper cell, Type 2 T helper cell, Activated dendritic cell, Eosinophil, Mast cell, MDSC, Monocyte, Natural killer cell, Natural killer T cell, Neutrophil, Plasmacytoid dendritic cell) between the low and high risk score groups.

### Analysis of SNP, CNV, TMB, MSI, and TIDE in the low and high riskscore groups

**FIGURE 6:**
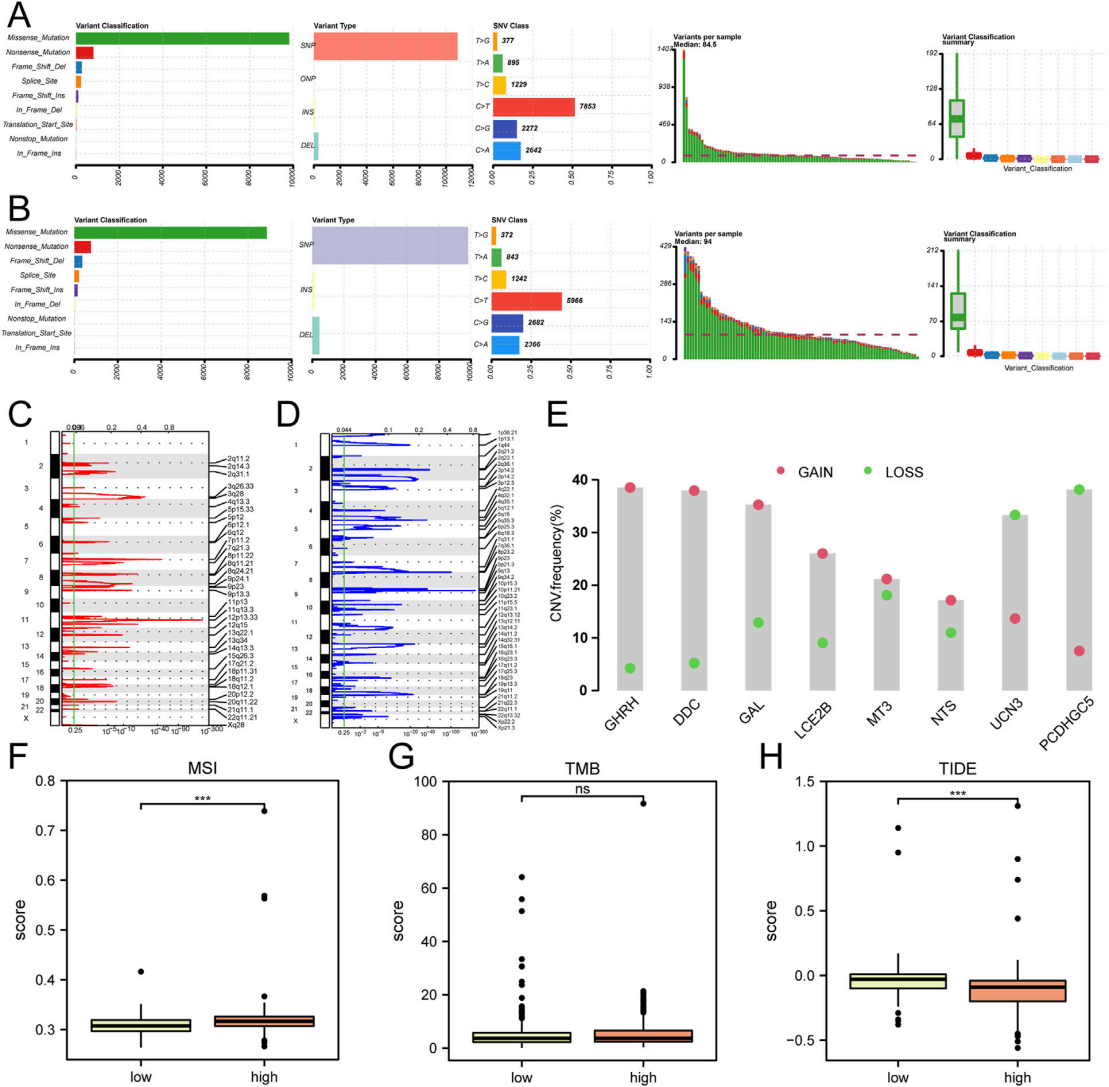
Analysis of SNP, CNV, TMB, MSI, and TIDE in the TCGA-HNSC Dataset **(A)** Comparative analysis of SNP in the Cox low riskscore group. **(B)** Comparative analysis of SNP in the Cox high riskscore group. **(C, D)** GISTIC analysis depicting copy number variations (CNVs) in HNSC samples from the TCGA dataset. The red color indicates increased CNVs **(C)**, while the blue color indicates decreased CNVs **(D)**. **(E)** CNV analysis of hub genes in the TCGA-HNSC dataset. **(F–H)** Comparative assessment of microsatellite instability (MSI) **(F)**, tumor mutation burden (TMB) **(G)**, and TIDE immune therapy scores **(H)** between the low and high riskscore groups identified through Cox regression analysis in the TCGA-HNSC dataset. ***P < 0.001.

The somatic mutation profiles of patients in the Cox regression analysis-based low-risk and high-risk score groups were examined in the TCGA-HNSC dataset. The findings revealed that the primary types of somatic mutations observed in both groups were Missense Mutation and Nonsense Mutation, with a higher prevalence of missense mutations. Additionally, single-nucleotide polymorphisms (SNPs) were identified as the most common type of mutation observed in HNSC patients within the Cox low-risk and high-risk score groups. In the low-risk score group, the most frequent single nucleotide variant (SNV) among HNSC patients was C > T, followed by C > A (Figure 6A). Conversely, in the high-risk score group, the most prevalent SNV among HNSC patients was C > T, followed by C > G (Figure 6B).

The Copy Number Variation (CNV) analysis demonstrated an increased number of CNVs at 11q13.3 (Figure 6C) and a decreased copy number at 9p21.3 in the mutant group of the TCGA-HNSC dataset (Figure 6D). Among the 8 hub genes analyzed for CNV, GHRH exhibited the highest amplification frequency, while PCDHGC5 had the highest deletion frequency (Figure 6E).

Furthermore, an evaluation of microsatellite instability (MSI) and tumor mutation burden (TMB) was conducted among the low-risk and high-risk score groups of HNSC patients based on Cox regression analysis in the TCGA-HNSC dataset. The sensitivity to immune therapy across different groups of HNSC patients was assessed using the TIDE algorithm. The analysis indicated no statistically significant differences in TMB between the various groups (Figure 6G). However, a statistically significant difference (P < 0.05) in MSI (Figure 6F) and TIDE scores (Figure 6H) was observed among the different groups of HNSC patients in the dataset.

### Construction of a clinical prediction model utilizing lactic acid metabolism-associated risk score

**FIGURE 7:**
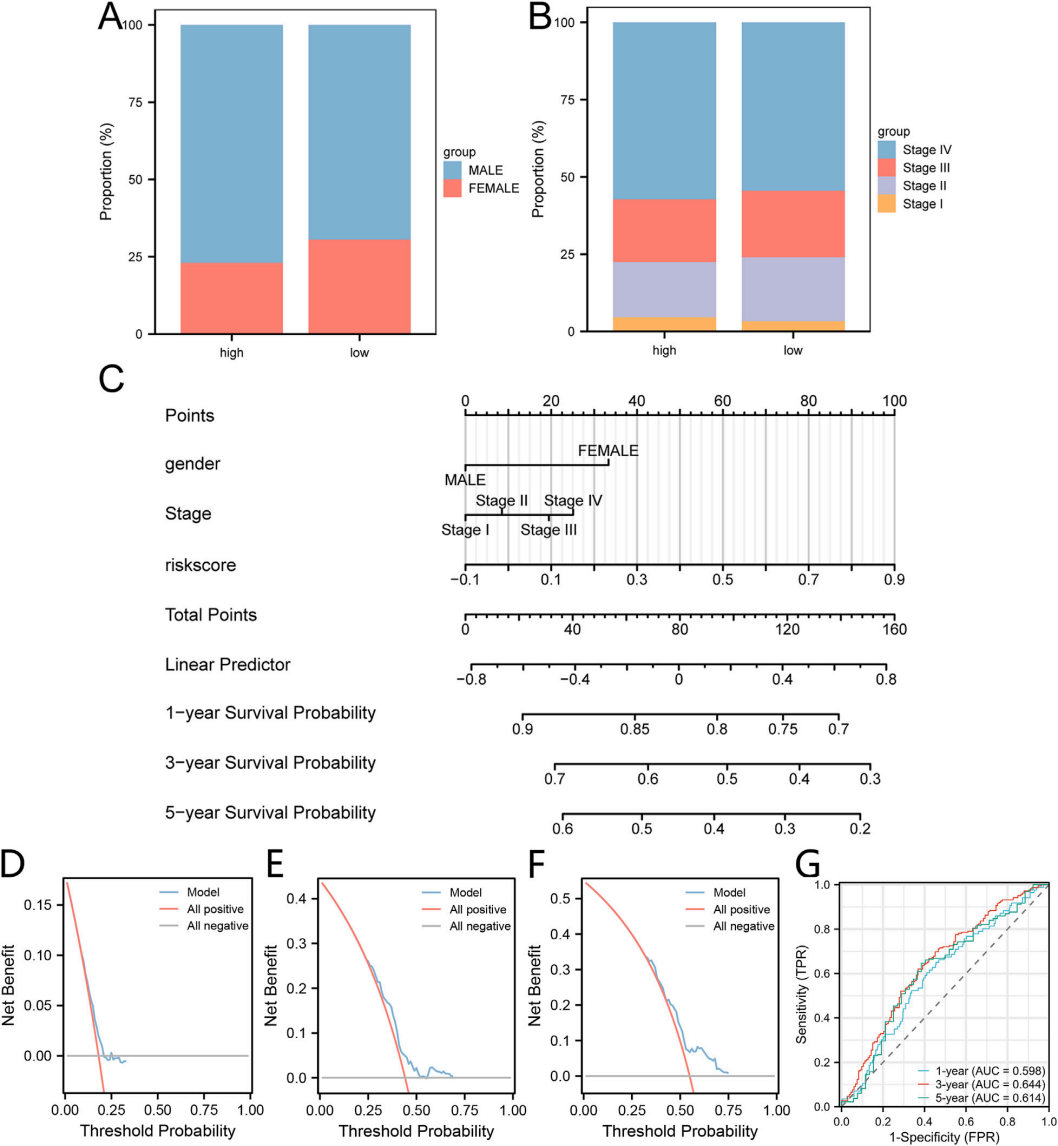
Construction of the clinical prediction model utilizing risk score associated with lactic acid metabolism. **(A, B)** The proportion of gender **(A)** and disease stage **(B)** within the high-group and low group is depicted in stacked bar charts. **(C)** A nomogram is presented, illustrating the integration of lactic acid metabolism-related risk score with clinical pathological features to construct the clinical prediction model. **(D–F)** Calibration curves are shown, representing the performance of the clinical prediction model at one-year **(D)**, three-year **(E)**, and five-year **(F)** intervals. **(G)** Time-dependent ROC curve illustrating the predictive ability the clinical prediction model at one-year, three-year, and five-year intervals.

To delve deeper into the clinical relevance of the risk score linked to lactic acid metabolism, a comparative analysis was conducted to examine the distribution of gender and disease stage between the high-risk and low-risk groups (Figures 7A, B). Subsequently, a prognostic model was constructed for patients with HNSC based on the risk score obtained from Cox regression analysis, which considered mitochondrial energy metabolism as well as clinical and pathological features including age and disease staging. The resulting model was visualized using a column line chart (nomogram) (Figure 7C). The accuracy of the model was evaluated using calibration curves, which demonstrated a strong agreement between the estimated one-year (Figure 7D), three-year (Figure 7E), and five-year (Figure 7F) overall survival (OS) values and the actual observations of patients. Additionally, the ROC curve (Figure 7G) showed that the mode had a certain predictive ability for the overall survival of HNSC patients.

### Validation of Hub Genes

**FIGURE 8:**
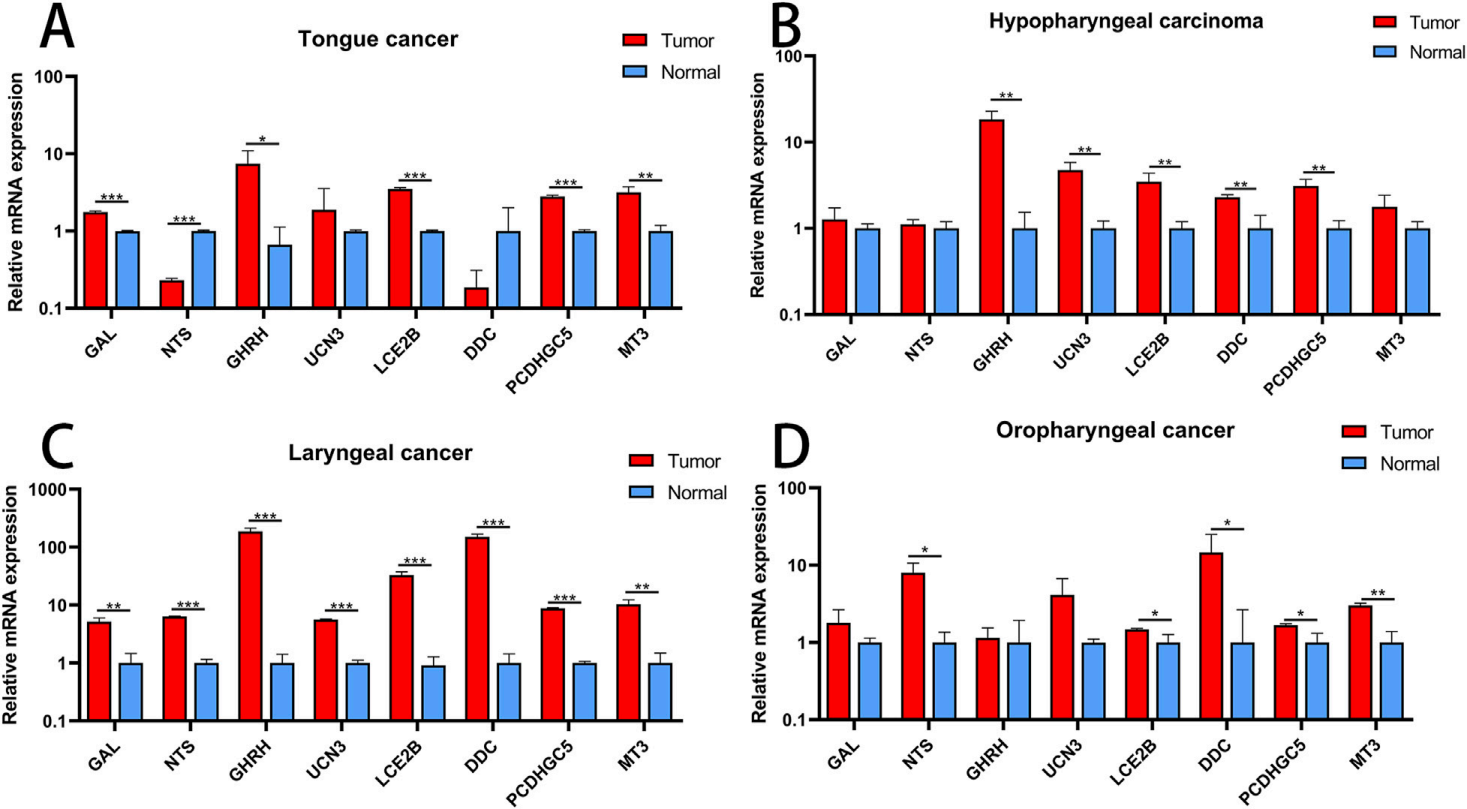
Validation of Hub Genes **(A)** Expression profiles of hub genes in tongue cancer. **(B)** Expression profiles of hub genes in hypopharyngeal cancer. **(C)** Expression profiles of hub genes in laryngeal cancer. **(D)** Expression profiles of hub genes in oropharyngeal cancer. *P < 0.05; **P < 0.01; ***P < 0.001.

The expression levels of eight hub genes were examined in different types of head and neck cancers, including tongue cancer, hypopharyngeal cancer, laryngeal cancer, and oropharyngeal cancer. The findings revealed that, in comparison to their levels in normal tissues, the genes GAL, GHRH, LCE2B, PCDHGC5, and MT3 were notably upregulated in tongue cancer, while NTS was downregulated. For hypopharyngeal cancer, GHRH, UCN3, LCE2B, DDC, and PCDHGC5 were upregulated. All eight key genes displayed upregulation in laryngeal cancer. Regarding oropharyngeal cancer, NTS, LCE2B, DDC, PCDHGC5, and MT3 demonstrated increased expression levels (Figure 8).

## Discussion

This study represents the first attempt to explore the correlation between LMRGs and HNSC. We conducted a comprehensive analysis of 289 LMRGs in HNSC patients and assessed their LMs. Based on the median LMs value, patients were categorized into LMs-high and LMs-low groups. Notably, a significant observation was made that patients in the LMs-high group exhibited a more unfavorable prognosis than those in the LMs-low group. Consistent with previous studies linking lactate metabolism to cancer invasion and metastasis, (Brand et al., 2016; Brown and Ganapathy, 2020; Bergers and Fendt, 2021), we observed a correlation between LMs and M stage and N stage. Specifically, all patients at the M1 stage were found in the LMs-high group, and a higher proportion of N2-stage patients were observed in the LMs-high group compared to the LMs-low group. Additionally, we identified key genes associated with prognosis in both the LMs-high and LMs-low patient groups, constructing a Cox regression prognostic model. Remarkably, HNSC patients with high-risk scores based on the Cox risk assessment exhibited significantly poorer prognoses. To assess the performance of the Cox risk score in predicting overall survival for HNSC patients, we employed decision curve analysis and ROC curves, both of which confirmed its excellent predictive capability. Moreover, the predictive effectiveness of the Cox risk score was shown to enhance with time.

Furthermore, we identified key genes that influence prognosis within the LMs-high and LMs-low patient cohorts and developed a Cox regression prognostic model based on these findings. It was observed that HNSC patients with higher risk scores through the Cox risk assessment exhibited poorer prognosis. To evaluate the predictive capacity of the Cox risk score for OS in HNSC patients, we employed DCA and ROC curves, both of which confirmed its robust predictive capacity. Consistent with previous research on lung adenocarcinoma (Zhao et al., 2022), the predictive performance of the Cox risk score improved over time.

In addition to the Cox risk score, we performed differential gene enrichment analysis using GSEA. This analysis revealed significant enrichment of genes involved in pathways such as Gluconeogenesis, Pyruvate metabolism, Cellular Response to Hypoxia, and PI3K/AKT Signaling in Cancer between the high-risk score and low-risk score groups identified by the Cox regression model. These findings support the association of the Warburg effect (Sato et al., 2016) and dysregulated PI3K/AKT signaling pathway with the metabolic switch towards aerobic glycolysis and lactate production (Gottlob et al., 2001).

In addition, we identified eight key hub genes (GAL, NTS, GHRH, UCN3, LCE2B, DDC, PCDHGC5, MT3) for further investigation. Galectins, a class of lectins, are known to be upregulated and play a crucial regulatory role in the development of various diseases (Kapetanakis and Busson, 2023). Numerous studies have demonstrated that dysregulation of metabolic disease-induced fibrosis promotes cancer progression by inducing Galectin expression within the tumor microenvironment (Rao and Rao, 2017; Li et al., 2021). Neurotensin (NTS) and its receptor NTSR1, a neuroendocrine peptide, primarily regulate tumor initiation, proliferation, apoptosis, metastasis, and differentiation through three pathways: the IP3/Ca2+/PKC/MAPKs pathway, the MMPs/EGFR/MAPKs (PI3K/Akt) pathway, or the Rho-GTPases and non-receptor tyrosine kinase pathway (Kim et al., 2015; Feng et al., 2024). Growth hormone-releasing hormone (GHRH) antagonists effectively inhibit the *in vivo* growth of various experimental cancers (Barabutis et al., 2018; Barabutis and Schally, 2010). Urocortin 3 (UCN3), a peptide hormone, is associated with cuproptosis and immunity in colon cancer (Huang et al., 2023). LCE2B regulates vascular lymphatic invasion and correlates with poor survival in laryngeal cancer (Metzger et al., 2022). The expression of DDC significantly correlates with an immunosuppressive tumor microenvironment, higher intra-tumoral heterogeneity, elevated expression of the immune checkpoint CD274, and possibly mediates malignant behaviors of ccRCC cells via the PI3K/Akt signaling pathway (Chang et al., 2022). PCDH10, a member of the non-clustered protocadherin family δ2 subtype, has recently been shown to inhibit the growth, migration, invasion, and colony formation of tumor cells, and may act as a tumor suppressor gene involved in tumorigenesis (Curia et al., 2019). The MT3 gene, belonging to the metallothionein gene family, encodes metallothioneins. Research has found that downregulation of MT3-MMP promotes tumorigenesis and correlates with poor prognosis in esophageal squamous cell carcinoma (Xue et al., 2016).

This study aimed to investigate the relationship between Cox risk scoring and infiltration of immune cells. The Cox regression analysis model revealed significant variations in the ESTIMATE Score, Immune Score, and Stromal Score between the high-risk score and low-risk score groups. It is important to highlight that lactate accumulation within the TME leads to acidification, which has been shown to impede various functions of immune cells (Li et al., 2022). In both the high-risk score and low-risk score groups, notable disparities in immune cell infiltration were observed across 21 different types of immune cells. This finding suggests that lactate metabolism plays an active role in the regulation of the tumor microenvironment. Cox risk scoring could potentially aid in uncovering tumor immune regulatory mechanisms and offer novel perspectives for future studies on the TME.

Previous studies have demonstrated the association between somatic mutations and tumor heterogeneity, as well as their influence on treatment response (Chanock, 2018; Dalgliesh and Futreal, 2007; Guan et al., 2023).

Our findings indicate that the most commonly observed mutations in HNSC include missense mutations, SNPs, and C>T mutations. Additionally,the SNV spectrum exhibited risk-specific biases: C > T transitions predominated in both groups, but the low-risk cohort showed a secondary C > A transversion, while the high-risk group displayed elevated C > G transversions. This divergence suggests differential mutagenic processes, potentially linked to extrinsic carcinogen exposure or intrinsic DNA repair deficiencies, which may influence tumor evolution and therapeutic vulnerabilities. CNVs have emerged as key contributors to individual genetic variations, somatic transformation, tumor progression, and metastasis (Zhang et al., 2020; Carron et al., 2022). In our analysis of the TCGA-HNSC dataset, we observed the highest increase in copy number at 11q13.3 and the greatest decrease at 9p21.3 among the mutation groups. Notably, among hub genes, GHRH amplification and PCDHGC5 deletion emerged as dominant events, implicating dysregulated growth signaling (GHRH) and loss of cell-cell adhesion (PCDHGC5) as potential drivers of risk stratification (Mao et al., 2024). These findings align with prior studies linking 11q13.3 amplifications to poor prognosis in head and neck malignancies. These discoveries shed light on the underlying mechanisms driving malignant progression in HNSC and offer potential avenues for diagnostic research. Furthermore, our study explored the sensitivity of HNSC patients in high-risk score and low-risk score groups to immunotherapy, revealing significant differences between the two groups. Patients in the high-risk score group may potentially benefit more from immunotherapy compared to those in the low-risk score group. Moreover, our study demonstrated that eight hub genes are significantly associated with most immune cells, suggesting that Cox risk scoring can serve as a novel immune indicator for HNSC treatment. These findings may provide references for further investigations into the mechanisms of LMRGs in HNSC.

Our study has several limitations. First, the lack of direct assessment of immunotherapy response in the patient cohort, despite the prognostic model’s correlation with immune characteristics and immune evasion markers. Second, the retrospective nature of TCGA data introduces potential confounding factors due to treatment heterogeneity, including variations in immunotherapy types and durations across patients. Third, the functional analyses rely on *in silico* methods without experimental validation of the lactate metabolism gene signature’s impact on immunotherapy response *in vivo*. Additionally, while the prognostic model highlights associations with the tumor microenvironment, integrating clinical immunotherapy outcome data and experimental validations could further clarify its clinical utility.

In summary, lactate metabolism plays a significant role in HNSC. Cox risk scoring, which is based on lactate metabolism, correlates with patient prognosis and immune cell infiltration. Furthermore, it can predict patient sensitivity to immunotherapy. The prognostic risk model centered around lactate metabolism offers a fresh perspective for future HNSC prognosis and immunotherapy studies.

## Materials and methods

### Data source

We sourced RNA-Seq transcriptomic data from The Cancer Genome Atlas (TCGA) database (https://portal.gdc.cancer.gov), comprising 535 tumor samples and 59 normal samples, for HNSC patients (Colaprico et al., 2016). Concurrently, the corresponding clinical data were retrieved from the UCSC Xena database (http://genome.ucsc.edu). A total of 289 genes associated with lactic acid metabolism (LMRGs) were collected from the GeneCards database (Stelzer et al., 2016) and Molecular Signatures Database (MSigDB) (Liberzon et al., 2015). To compare the differences in tumor immune therapy between high-risk score and low-risk score groups, we employed the Tumor Immune Dysfunction and Exclusion (TIDE) algorithm (http://tide.dfci.harvard.edu) based on the Cox regression risk score. The copy number variation (CNV) segments underwent GISTIC 2.0 analysis using the Hiplot website (https://hiplot-academic.com/advance/gistic2). For the visualization of the protein-protein interaction (PPI) network and selecting hub genes, we utilized the Cytoscape software and the String website (Gao et al., 2013).

### Selection of key genes related to prognosis-associated LMRGs

To assess the prognostic relevance of the Lactic Acid Metabolism Score (LMs), we utilized the R package GSVA and ssGSEA algorithm to calculate LMs based on the expression matrix of LMRGs (Hänzelmann et al., 2013). In order to categorize patients into high and low risk groups based on the expression of the five key genes, we developed a prognostic risk model using Cox regression analysis. This model incorporates the expression levels of all five genes to generate a risk score for each patient. Patients were then divided into two groups based on the median risk score: those with a risk score above the median were classified as the high-risk group, while those below the median were classified as the low-risk group. Next, we identified Differentially Expressed Genes (DEGs) associated with the high-LMs and low-LMs groups in the TCGA-HNSC dataset by employing the DESeq2 R package, adhering to stringent criteria of |logFC|>0.5 and p.adjust<0.0001 (Love et al., 2014). Furthermore, we obtained co-expression modules linked to the high-LMs and low-LMs groups using the WGCNA package (Zhang and Horvath, 2005). The pivotal genes were identified by intersecting the DEGs with LMRGs and those genes demonstrated the highest correlation with LMs within the co-expression modules. Finally, univariate Cox regression analysis was conducted to pinpoint key genes that were significantly associated with patient prognosis.

### Consensus clustering analysis of prognosis-associated key genes

Key genes predictive of prognosis were identified through univariate Cox regression analysis. The validation of key gene expression on prognosis was conducted by scrutinizing Kaplan-Meier (KM) survival curves. Subsequently, patients were categorized into distinct molecular subtypes based on the prognostic key genes, employing the Consensus Cluster Plus R package. To analyze the data, Principal Component Analysis (PCA) was performed using the “ggplot2” R package (Ringnér, 2008).

### Differential gene selection based on Cox multifactorial model risk stratification

The expression profiles of key genes were interrogated through the application of the Cox regression model. From the Cox multifactorial model, the top 20 differentially expressed genes (DEGs) associated with high and low-risk score groups were selected for further analysis. The GOSemSim package computed the top 20 DEGs linked to risk stratification in the Cox multifactorial model. Subsequently, the protein-protein interaction (PPI) network for these top 20 DEGs was constructed utilizing the String website, while the mRNA-miRNA interaction network was established using the MiRDB database (Gao et al., 2013).

### GSEA enrichment analysis

The clusterProfiler package was employed to perform enrichment analysis on the DEGs. The gene set “c2.cp.all.v2022.1.Hs.symbols.gmt [All Canonical Pathways] (3050)” was acquired from the MSigDB database. Significantly enriched pathways were chosen based on stringent criteria, including a p.adjust <0.05 and a false discovery rate (FDR) value (q.value) < 0.25 (Liberzon et al., 2015).

### Integrated analysis based on Cox multifactorial model risk stratification in HNSC

The estimate package was utilized to assess the differences in the immune score, stromal score, and ESTIMATE score between the high-risk score and low-risk score groups, as determined by the Cox multivariate model employing the ESTIMATE algorithm. The infiltration levels of immune cells in each sample were analyzed using Single-sample Gene Set Enrichment Analysis (ssGSEA) (Barbie et al., 2009). For the analysis of gene mutations in HNSC samples derived from the TCGA database, the “MAFTOOLS” R package was employed (Mayakonda et al., 2018). Additionally, copy number variation analysis of HNSC samples from the TCGA database was carried out using the “TCGAbiolinks” R package (Mermel et al., 2011).

### Construction of clinical model based on Cox multivariate risk score

To evaluate the predictive efficacy of overall survival (OS), the Cox multivariate risk score was combined with patients’ clinical and pathological characteristics. Subsequently, a clinical prediction nomogram was constructed by incorporating the risk score model and clinical and pathological features. The performance of the nomogram was assessed by comparing the predicted values from the nomogram with the observed survival rates, which generated calibration curves. The calculation formula for the risk score is as follows:

$$\text{Risk}Score=\sum_{i}Coefficient\;\left({gene}_{i}\right)*mRNA\;Expression\;\left({gene}_{i}\right)$$

### RT-qPCR

TRK Lysis Buffer (20–30 mg tissue/700 µL TRK Lysis Buffer) was used to extract total RNA from 12 pairs of HNSC tumors and adjacent tissues. All of the specimens were approved by the Research Ethics Committee of the Third Xiangya Hospital of Central South University (XMXH-2022-3282). Following the instructions of the ReverTra Ace qPCR RT Kit, cDNA synthesis was carried out using the Enzyme Mix containing reverse transcriptase and the Primer Mix. KOD SYBR^®^ qPCR Mix was utilized for RT-qPCR. Data analysis was performed using the 2^−ΔΔCT^ values. The primer sequences for the hub genes are detailed in Supplementary Table 1.

### Statistical analysis

The previous section has presented an extensive explanation of the statistical methods employed in processing transcriptome data. R software (Version 4.1.2) was utilized to perform all statistical analyses, with a significance level set at p < 0.05.

## Data Availability

The datasets presented in this study can be found in online repositories. The names of the repository/repositories and accession number(s) can be found below: https://www.ncbi.nlm.nih.gov/, GSE58911 https://www.ncbi.nlm.nih.gov/, GSE83519 https://www.ncbi.nlm.nih.gov/, TCGA-HNSC.

## Supplementary material

SUPPLEMENTARY TABLE 1 The primer sequences for the hub genes.

## Abbreviations

HNSC, head and neck squamous cell; LMs, Lactate Metabolism score; LMRGs, Lactate Metabolism-related genes; WGCNA, Weighted gene co-expression network analysis; PPI, Protein-protein interaction; GO Gene Ontology; KEGG, Kyoto Encyclopedia of Genes and Genomes; GSEA, Gene Set Enrichment Analysis; SNP, single nucleotide polymorphism; CNV, Copy number variation.
