# Supplementary figures and images for: The gene signature linked to lactate metabolism predicts the prognosis and correlates with the immune status of head and neck squamous cell carcinoma
Source: Front Genet. 2025 Apr 4;16:1540841. doi: 10.3389/fgene.2025.1540841 (PMC12006151; doi:10.3389/fgene.2025.1540841)

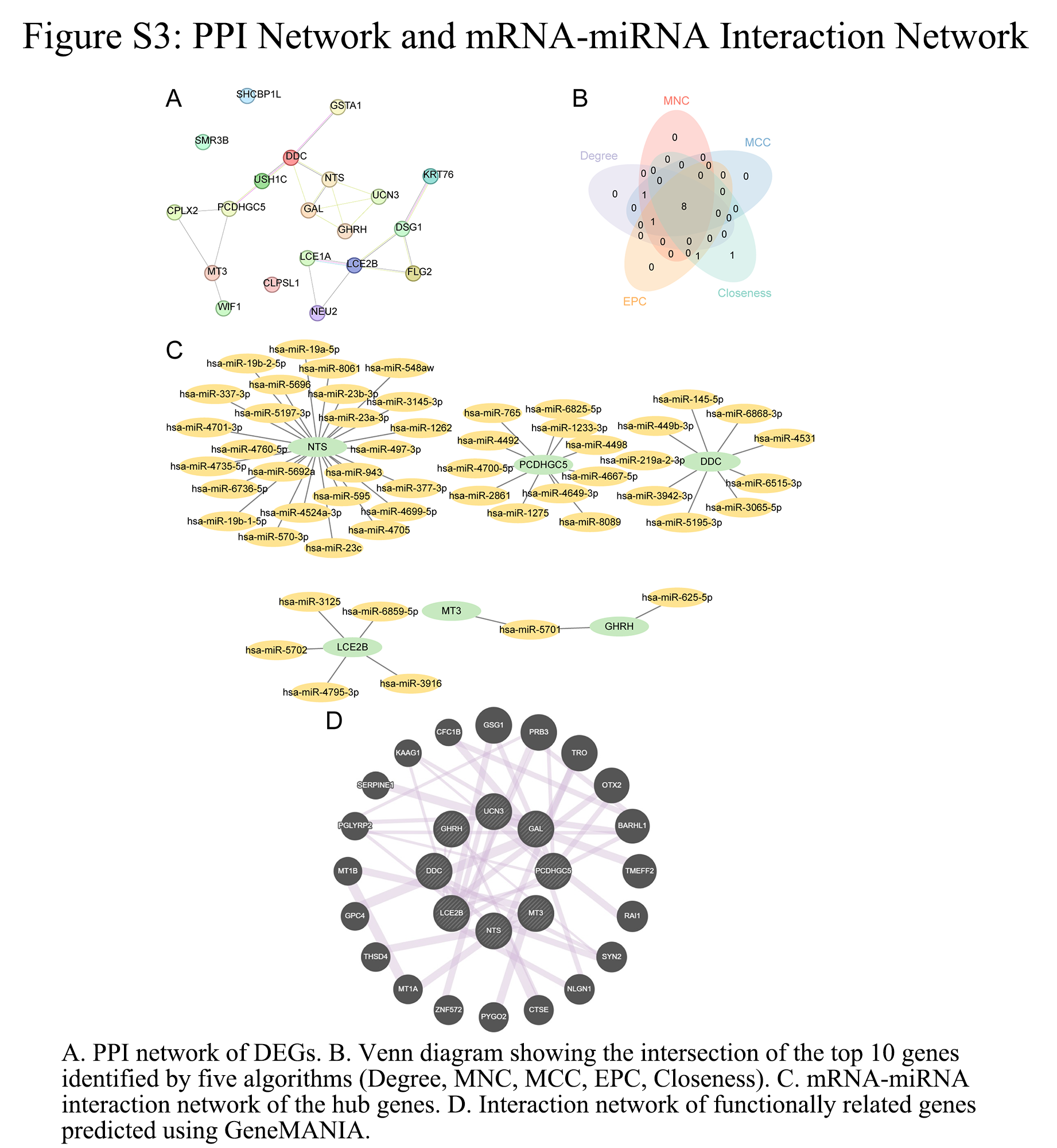

Supplement: Supplementary file 2 [file Image3.tif]

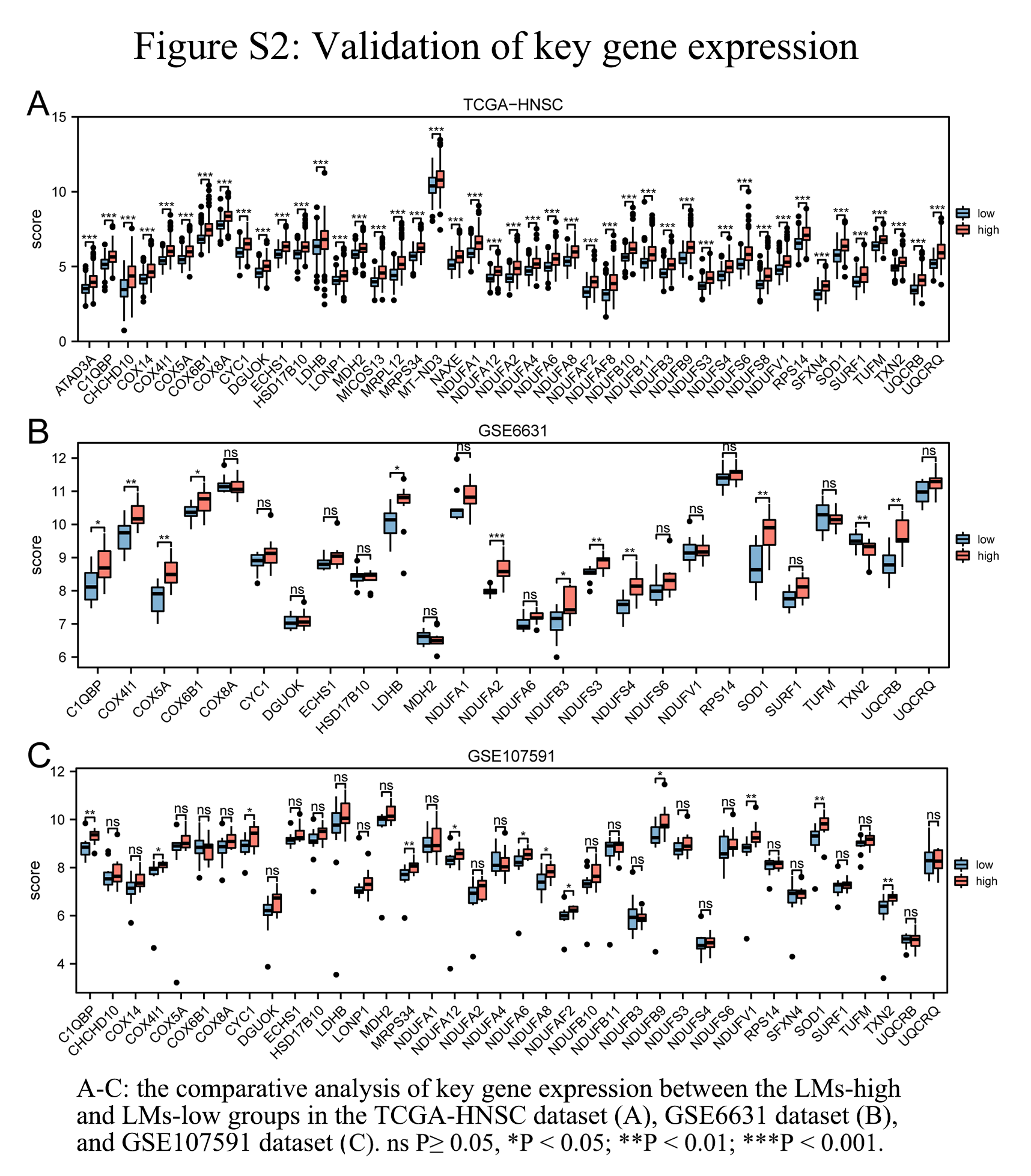

Supplement: Supplementary file 3 [file Image2.tif]

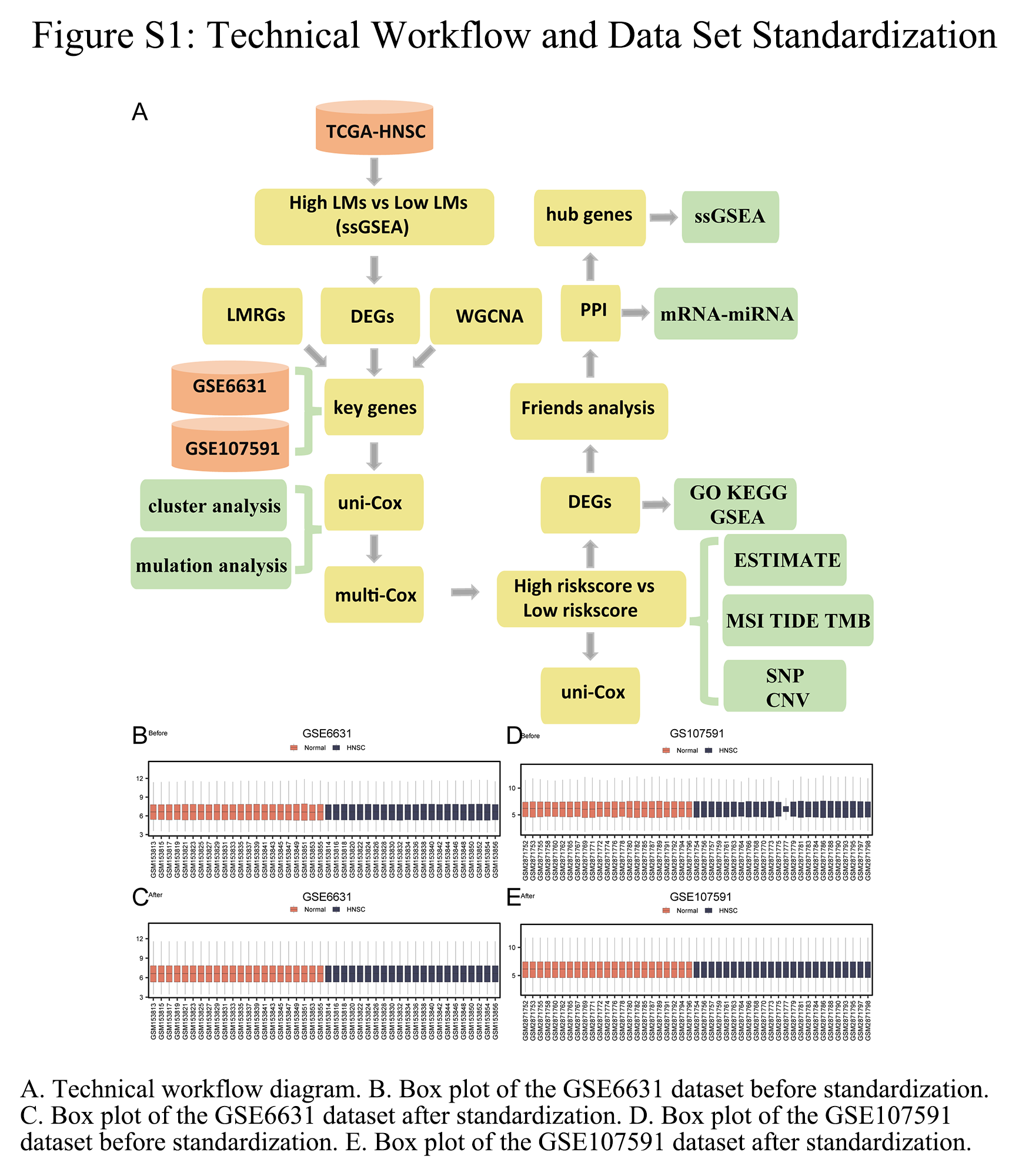

Supplement: Supplementary file 4 [file Image1.tif]
